# Footwear fit in schoolchildren of southern Spain: a population study

**DOI:** 10.1186/s12891-019-2591-3

**Published:** 2019-05-10

**Authors:** María Luisa González Elena, Antonio Córdoba-Fernández

**Affiliations:** Departamento de Podología, Universidad de Sevilla, Calle Avicena s/n, 41009 Sevilla, Spain

**Keywords:** Foot, Fit, Footwear, Schoolchildren

## Abstract

**Background:**

Recent studies support the theory that ill-fitting shoes are an important source of pain and may lead to foot malformations in the medium term. Taking as reference the ideal allowance considered in the literature, the purpose of this study was to verify the outdoor footwear fit in a population of schoolchildren of southern Spain.

**Methods:**

Five hundred and five children within the range of 3–12 years of age participated in this study. Using a measuring instrument designed and validated for this purpose, maximum foot length, width and height were obtained from the longest foot. These measurements were compared with the inner length, width and height of the footwear. An adequate toe allowance of 5–15 mm in length and 10 mm in width were estimated.

**Results:**

Inner footwear length was shorter than foot length in 33.3% of the schoolchildren. Based on the allowance established, it was observed that the footwear of the schoolchildren was too short and too narrow in 72.5 and 66.7% of the cases, respectively.

**Conclusions:**

Only one third of the participants analysed had well-fitted footwear. The results show that it is necessary to raise awareness among parents and teachers about the importance of replacing, periodically, the footwear of children in primary education. Manufacturers should adapt the lasts considering the use of 90th percentiles instead of mean values obtained from the growth curves of schoolchildren’s feet.

## Background

The literature about the association between footwear and children’s foot health is confusing and full of contradictory findings. Some recent studies strongly support the theory that ill-fitting shoes are an important cause of foot pain and the consequent emergence of malformations related to this [1]. Existing meta-analyses show that the impact of footwear on gait should be considered when assessing the paediatric patient and evaluating the effect of shoe or in-shoe interventions [2]. Observational studies carried out in this field have shown a high growth rate in the foot length of schoolchildren. A recent study conducted in German schoolchildren between 6 and 14 years of age, determined an average foot growth in length of 6.2 mm/ year in boys and 4.2 mm/year in girls [3]. In the same way, the current evidence shows that during the child’s development and until the age of 8 years, the foot predominantly grows in length and after this age, the width/length proportion in older children is similar to observed in adults [4].

These rapid changes may be conditioned by extrinsic factors, such as footwear, or intrinsic factors, such as foot morphology or body mass index, and which in combination, can influence the morphological and functional development of the adult foot. With these growth rates, it seems only logical that the footwear of schoolchildren may become small quickly. Current reviews on the topic emphasize the importance of taking into account the changing morphology of children’s feet, which, along with their high functional demand, must be considered when designing ergonomic footwear [5, 6]. In the same way, some studies have shown that foot shapes differ between populations and genders, and for proper footwear fit, footwear should have enough size varieties to imitate different foot shapes of all humanity [7, 8].

There are very few studies focused on analysing the ideal toe allowance in length and width of children’s footwear. Some authors consider that, for a shoe to fit properly in length, there should be a minimum toe allowance (TA) of 5–12 mm between the tip of the shoe and the longest toe in the footwear of schoolchildren until the age of 16 years [3]. This allowance takes into account the dynamic increases that occur in the size of schoolchildren’s feet, which may range between 2.1 and 4.4 mm in length and reach around 2 mm in width, according to previous studies [9, 10]. Müller et al. found that most static and dynamic foot characteristics change continuously during growth and maturation, and these changes should be taken into account when development of suitable children’s shoes [4]. Even though footwear width allowances have not been established in a precise manner, the authors recommend a sufficient allowance, since the forefoot is especially sensitive to external influence [3, 11, 12]. The main purpose of this study was to verify the outdoor footwear fit in a schoolchildren population of southern Spain, taking as reference the ideal toe allowance considered in the literature.

## Methods

### Study design and participants

A prospective transversal study was designed with the aim of analysing the outdoor footwear fit of a sample of schoolchildren in length, width and height. Through a purposive sampling and using as the selection criterion the geographical proximity to the education centre where the study was carried out, three centres from the city of Sevilla (Spain) were selected. The randomized sample included one Early Childhood Education centre and two Early Childhood and Primary Education centres. For the samples election, a probabilistic sampling was used from a study population composed of 726 schoolchildren. With the consent of their parents or guardians, schoolchildren between 3 and 12 years of age were selected, who did not have any malformations or previous history of foot surgery. The children who wore boots or high-top footwear on the day of examination were excluded from the sample. The final study sample consisted of 505 schoolchildren. The study was carried out from February to May 2015, within the framework of a school health program, and it was approved by the Research Ethics Committee of the Universidad of Sevilla (Sevilla, Spain).

### Measurements

**Fig. 1:**
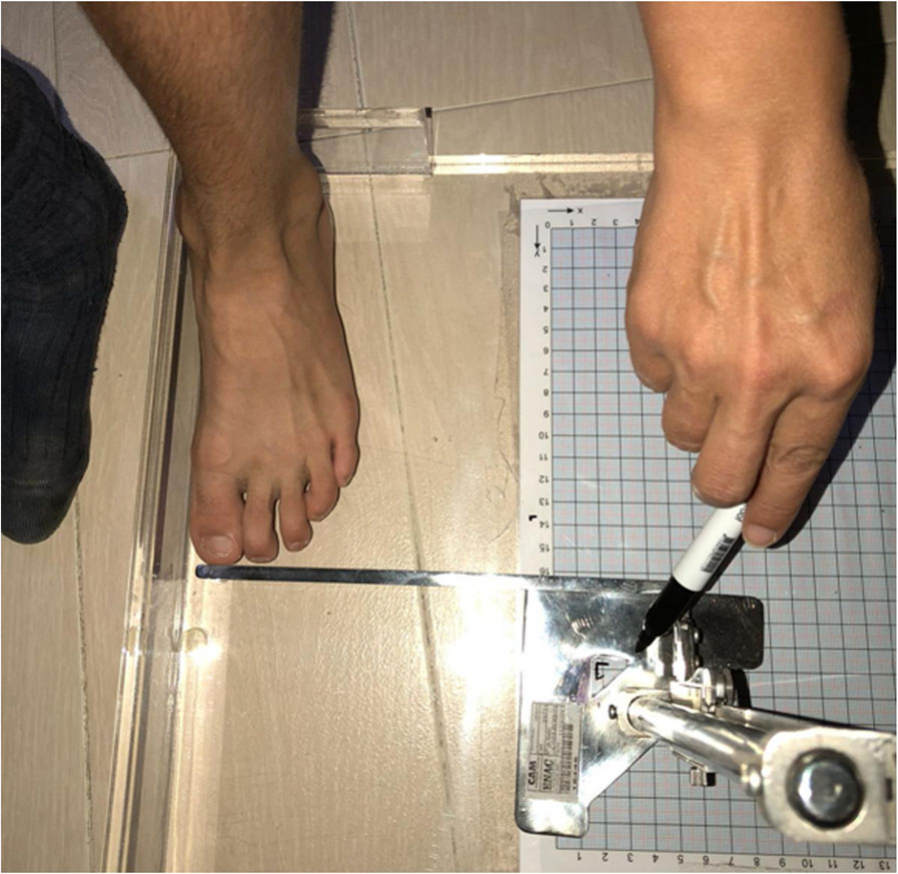
Acetate sheet placed on a methacrylate base with a calibrated template imprinted for FL and FW measurements

A measuring device was designed to determine the maximum foot length (FL), foot width (FW) and height at the level of the first metatarsal head (FH), and transfer the measurements to the inside of the shoe, in order to verify its fit. To carry out the measurements, the longest foot was selected, which was determined by measuring both feet separately from the heel to the longest toe using a retractable measuring tape while the participant was standing. When the measurement obtained was identical for both feet, the foot to be studied was randomly selected by flipping a coin in the air (heads = left foot, tails = right foot). To make the measurements traceable, the participants stood barefoot, with both feet at the same height and their knees extended, on an acetate sheet placed on a methacrylate base, which had a calibrated template imprinted in it (Andalusian Centre of Metrology; model CAM-V-00014-GRID-POD-03). This base had two protruding surfaces that made up a 90° angle at one of its corners, where the heel and the medial or lateral edge of the study foot were placed. Using a digital height gauge with a scribe marker (Andalusian Centre of Metrology; model CAM-PDVC-150-GRAMILPOD-01), it was possible to transfer the relative position of the reference points taken from the foot to the acetate sheet placed on the Grid (one point for the FL and two for the WL) (Fig. 1). The length was obtained directly by transparency through the scale of the Grid and the width was obtained from the X and Y 115 coordinates of each point using Access 11.0 (Microsoft Office 2010) software.

Unlike the measuring devices used in other studies [11, 13], our measurement took into account the maximum length of the foot according to the digital formula of the schoolchildren. To achieve this, we considered three different digital formulas, Egyptian foot formula (first longest toe), Greek foot formula (second longest toe) or square foot formula (first and second toe of the same length). To perform the measurement, the interior capacity of the footwear was taken into account taking as reference the maximum foot length. The maximum length of the foot (FL) was defined as the distance from the posterior side of the heel to the end of the longest toe [14]. The maximum metatarsal width (FW) was considering as the distance from the most protuberant medial and lateral points corresponding to the head of the first and fifth metatarsal bones [9, 13]. The maximum height (HL) was defined as the distance from the floor to the highest area of the first metatarsal head [15]. Measurements were precise to 0.01 mm. All the measurements were taken at schools by two operators.

The inter-observer reliability of the researchers and the measurements were calculated with an interval of 1 week. Inter-observer intraclass correlation coefficient (ICC) with level of confidence of 95% (Cronbach’s α) was 0.99 for FL, 0.98 for FW, 0.99 for footwear length (FWL), and 0.99 for footwear width (FWW). Coefficient of variation (CV) was 13.15% for FL, 12.59% for FW, 13.08% for FWL, and 9.74% for FWW. Inter-observer relative technical error of measurement (TEM %) was 0.20, 0.07, 0.21, and 0.06 respectively.

**Fig. 2:**
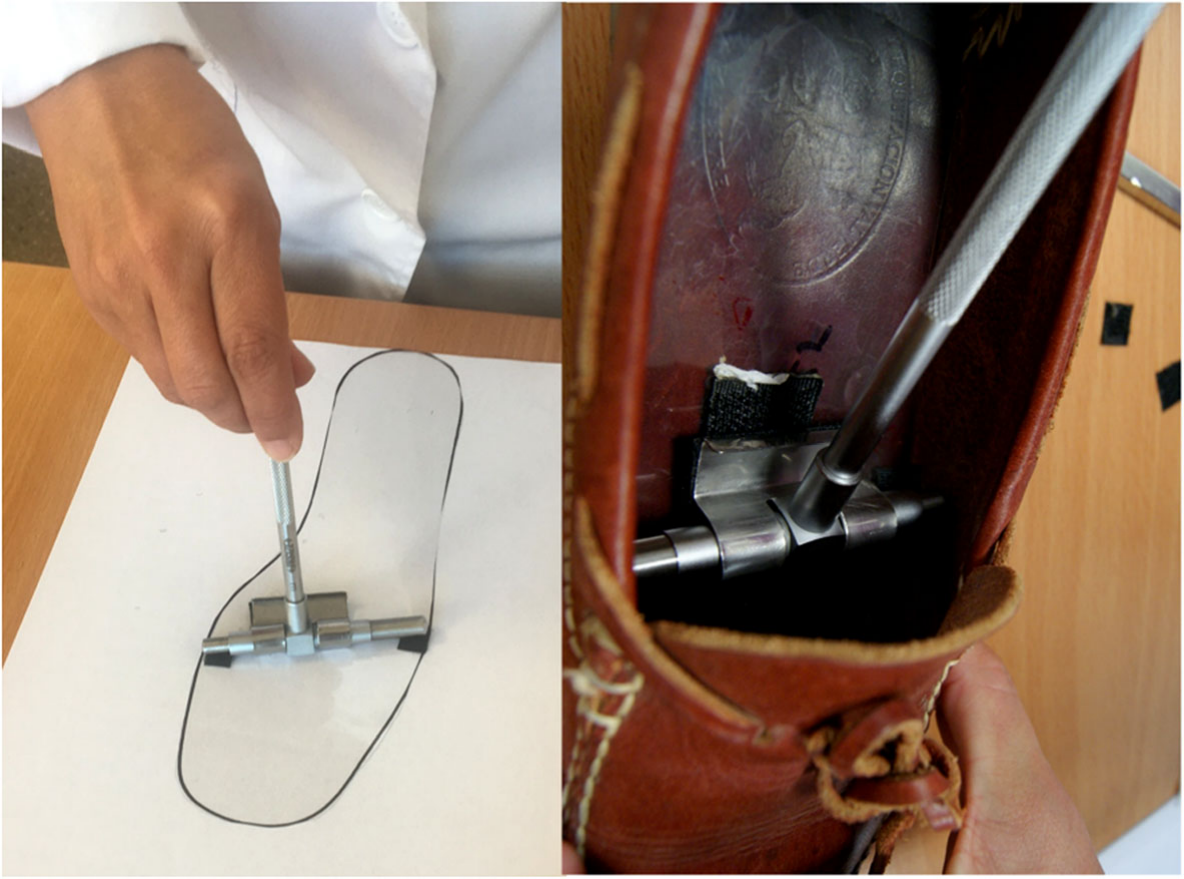
The image on the left shows acetate transparent insole with the width reference points. The image on the right shows telescopic gauge inside of the footwear

**Fig. 3:**
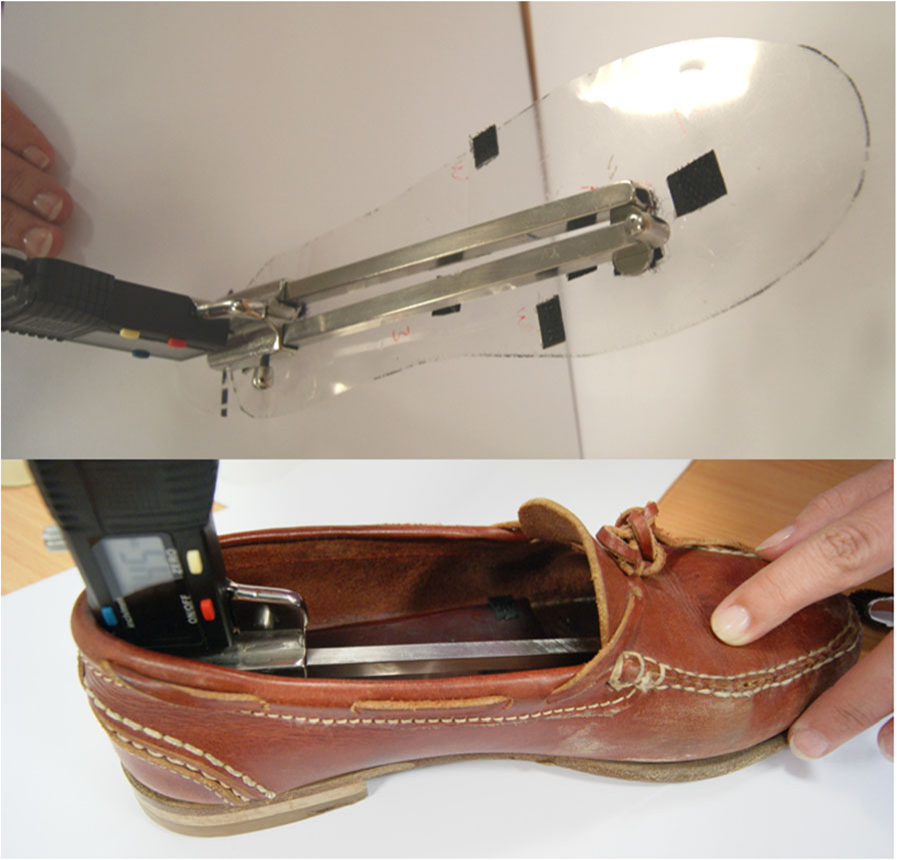
Telescopic gauges with protractors used to check the footwear fit in height

Once these measurements were obtained, they were transferred to the inside of the footwear using a acetate transparent insole that had approximately the same size as that of the foot of the schoolchild, where the length, width and height reference points obtained were placed using a hook-type Velcro surface tape. In order to calculate the length, width and height of the inside of the footwear, telescopic gauges with protractors were used (Andalusian Centre of Metrology; interior gauge, model CAM-0-150 mm-POD-02). Using a loop-type Velcro surface tape, the gauge was fitted to the insole at the points marked with hook-type Velcro surface tape and it was then inserted into the shoe (Figs. 2 and 3). The gauge had a brake that could be released once inside the footwear, which allow edit to expand longitudinally, transversally and upwards until it met the inner edges of the shoe; at this point, the break was activated again and, once the gauge was extracted from the shoe, it was possible to accurately determine the length, width and height of the footwear using a retractable measuring tape. Foot measurements were obtained from the longest foot, and were compared with the inner length, width and height of the footwear.

The expansion of the measuring device lengthwise was determined by the resistance generated by the material of the heel counter and of the reinforcement of the toe tip. For the width and height measurements, the position of the touch probes when they reach the measuring points without deforming the material was taken into account. An adequate toe allowance (TA) of 5–15 mm in length and 10 mm in width was estimated.

### Statistical analysis

The statistical analysis was conducted using the statistics software IBM SPSS v22 (SPSS, Inc., Chicago, IL, USA). To determine the three-dimensional fit of the footwear, the difference between the shoe and foot measurements of length, width and height was calculated. This way, a positive result showed that the footwear was longer or wider than the foot and, on the contrary, a negative result indicated that the footwear was shorter or narrower. If the result was zero, it meant that there were no differences between the dimensions of the footwear and those of the foot. Based on the TA considered, it was established for the present study that the footwear was well-fitted when the distance between the toecap and the longest toe was between 5 and 15 mm. In the same way, the footwear was considered narrow when the space for the expansion of the foot in width was less than 10 mm. To compare the length and width fit, the Wilcoxon signed-rank test for related samples was used. To compare the length, width and height fit as a function of gender, the Mann-Whitney U-test was used. For the inferential analysis, a 95% confidence interval was considered; thereby, the experimental *p*-value was compared with a 5% significance level.

## Results

The total sample of individuals analysed was 505 schoolchildren (256 boys; 249 girls) with an average age of 6.79 ± 2.63 years. Distribution according to age group is shown in Table 1. With regard to the digital formula, 26.4% had Square feet, 34.5% had Egyptian feet and 39.1% had Greek feet. No significant differences were found regarding the digital formula according to gender. Likewise, no significant differences were observed with respect to shoe fit in length or width regarding the digital formula in those cases in which the footwear was too short or narrow.

**Table 1:** Characteristics of the sample. Mean and standard deviation of anthropometric variables for different age groups and the whole sample

Age (years)	N	Gender	Foot Length (mm)	Foot Width (mm)	Foot Height (mm)
3	47	f: 19; m: 28	175.59 ± 19.99	66.48 ± 0.63	21.33 ± 4.01
4	78	f: 34; m: 44	176.92 ± 15.10	69.81 ± 0.57	23.12 ± 3.75
5	79	f: 36; m: 43	186.73 ± 18.04	73.63 ± 0.64	24.72 ± 3.54
6	52	f: 27; m: 25	195.04 ± 16.76	74.14 ± 0.66	25.29 ± 4.00
7	53	f: 28; m: 25	209.60 ± 21.72	77.64 ± 0.68	27.92 ± 5.42
8	43	f: 25; m: 18	224.64 ± 22.94	78.09 ± 0.74	29.12 ± 4.78
9	45	f: 24; m: 21	227.97 ± 15.67	82.04 ± 0.59	29.02 ± 3.10
10	51	f: 29; m: 22	235.76 ± 18.91	84.31 ± 0.78	29.63 ± 4.50
11	45	f: 20; m: 25	246.30 ± 12.59	86.00 ± 0.56	31.58 ± 3.07
12	12	f: 7; m: 5	252.66 ± 15.94	86.67 ± 0.57	32.00 ± 3.35
3–12	505	f: 249; m: 256	207.43 ± 30.34	76.75 ± 0.88	26.60 ± 5.12

*N* number of participants, *f* female participants, *m* male participants

The left foot was longer than the right foot in 72.6% of the schoolchildren. The FL average in the age range of 5 to 12 years was 9.22 ± 3.68 mm in boys and 9.62 ± 8.17 mm in girls (*P* > 0.05). Highest FL average increase in girls occurred between 7 and 8 years. The FW average in the age range of 5 to 12 years was 0.22 ± 0.09 mm in boys and 0.18 ± 0.006 mm in girls (*P* > 0.05). The boys had wider feet than the girls with significant differences from 9 years old.

For all the sizes analysed, it was observed that the inner length of the footwear was greater than that indicated by the footwear manufacturers, with significant differences in 6 out of 16 sizes analysed. With respect to the length of the inside of the footwear compared to the foot, this was longer in 59.2% of the individuals, shorter in 33.3% and similar in 7.5%. Based on the established TA (5–15 mm), the footwear of 27.5% of the schoolchildren studied was well-fitted in length. The contrast analyses showed significant differences between the median of the FL and that of the footwear (*P* = 0.001) (Table 2).

**Table 2:** Comparison of the medians between foot length and footwear length

Length (mm)	Foot Length	Footwear Length	*P* Value
Median (minimun/maximun)	206 (140/299)	210 (119/298)	
Mean ± SD	207.43 ± 30.34	211.25 ± 33.47	
Tipical mean error	1.37	1.49	0.001 *
95% CI (Lower limit, Upper limit)	204.35–209.74	208.28–214.13	

Wilcoxon test*

With regard to width, the results obtained showed positive differences in 64.3% of the schoolchildren and negative differences in 7.9%, whereas in 27.5% of cases the width of the footwear and that of the metatarsus were the same. The FH recorded was greater than that of the footwear in 44.1% of the participants, and no differences were observed in 8.9% of the cases. The contrast analyses showed significant differences between the median of the FW and that of the footwear width (*P* < 0.001) (Table 3).

**Table 3:** Comparison of the medians between foot width and footwear width

Width (mm)	Foot Width	Footwear Width	*P* Value
Median (minimun/maximun)	80 (60/100)	80 (50/110)	
Mean ± SD	76.75 ± 8.83	85.70 ± 12.13	
Tipical mean error	0.41	0.54	0.001 *
95% CI (Lower limit, Upper limit)	75.30–76.90	84.60–86.80	

Wilcoxon test*

Considering the ideal allowances, it was observed that 72.5% of the schoolchildren used short footwear and 66.7% used narrow footwear. The Wilcoxon test for related samples showed that, in the age range between 7 and 11 years, the footwear was shorter than the foot, with significant differences. Likewise, in the age range between 4 and 9 years, the footwear was narrower than the foot, with significant differences. The Mann-Whitney U test did not show significant differences with respect to length and width according to gender.

## Discussion

Most authors agree in pointing out that children are more vulnerable to the effects of ill-fitting shoes. However, since there are numerous factors that influence foot morphology, it is difficult to ensure a correct fit between footwear and feet, unless footwear manufacturers pay special attention to this when designing shoes for schoolchildren. Considering the ideal TA, the results of the present study show that 72.5% of the children and preadolescents wore ill-fitting shoes when looking at the length of school shoes compared to the length of the feet. A cross-sectional study carried out in 858 Austrian preschool children of 3–6.5 years showed similar results, and observed that the outdoor footwear was shorter than the feet in 69.4% of the sample; they also reported a significant relationship between this occurrence and the risk of suffering from hallux valgus [11]. Similar results have been reported in a recent study conducted in South African children who found that 67% of the children and adolescents wore ill-fitting shoes shorter than the feet [16].

The results of our study are especially striking given that the ideal TA in length considered by other authors for the 90th percentile was less than 15 mm. Mean TA values obtained by Barisch-Fritz et al. were 5.0 ± 3.7 mm for girls and 6.9 ± 3.8 mm for boys participants. The 90th percentile of TA was 9.8 mm for female and 11.5 mm for male participants aged 6–14 [3].

We believe that TA is especially relevant considering that there are studies that associated too-short footwear and forefoot malformations [11]. It was not possible to compare the results of the present study in terms of footwear fit width and length, since no studies were found in the literature to analyse these parameters in schoolchildren. Even though there are no data regarding the necessary allowance in height for schoolchildren’s footwear, the results of some similar studies show the need to carry out periodic changes that favour such fit [3, 11, 12].

Interestingly, it was observed that the inner footwear length was greater than that indicated by the footwear manufacturers in all the sizes analysed. Although this occurrence may be considered an advantage, it is recommended to purchase footwear according to the size of the feet and not to the size of the shoe indicated by the footwear manufacturers.

Previous studies show that footwear manufacturers do not take into account the anthropometric differences related to the digital formula. Current shoe designs do not allow for the comprehensive 3-D foot shape, which means they are unable to reproduce the wide variability in foot morphology [8]. The general procedure used to design children’s footwear is on a linear scale, from lasts taken in adult feet with a square forefoot morphotype. These lasts constitute the model for the production of different sizes of children’s footwear. These same authors emphasize the fact that most footwear manufacturers companies do not change the size of the lasts to include feet with different digital formulae [7]. We have not found significant differences regarding footwear fit in length and width with respect to digital formula. It was observed that the square foot was the one that was best fitted to the tip of the shoe; however, we consider that when designing ergonomic footwear for schoolchildren, not only the different digital formulae must be taken into account, but also the position and orientation of the forefoot inside the shoe and its interaction with the shoe tip.

With respect to width, the results of the present study show that 66.7% of the children and preadolescents wore ill-fitting shoes too narrow. Most shoemaking systems do not consider this parameter when producing children’s footwear and the shoes available in the market do not always comprise different widths for the same length. On the other hand, the width considered when designing children’s footwear is that of the resting foot, even though the evidence shows that both length and width are greater in the weight-bearing foot, and even greater when moving. However, we consider that, although the existence of different widths for the same size would be ideal, it cannot be asserted categorically that it is necessary to produce different widths for the same length, since the differences observed in the present study were more homogenous for width than for length. It was not possible to compare the results of the present study in terms of footwear fit in height, since no studies were found in the literature to analyse this parameter in footwear of schoolchildren. Regarding gender, the variations in foot measurements underwent a gradual increase with age in both boys and girls. Although we have not found significant differences with respect to FL, as in similar studies carried out in our country, we have also found gender differences with regard to the FL average appeared at the age of 8–9 years with a greater FL of the girls [17]. Some studies have shown that the change foot size in length that occurs in North American girls ages 6 to 7 may represent an early marker for the transition to puberty [18]. Regarding the FW, and coinciding with recent studies, measurements showed that the boys had significantly wider feet than the girls in all of the age categories, although we have observed significant differences from 9 years old. Unlike this study, we have not found significant differences between boys and girls with regards to foot and shoe width [16]. The results of the present study demonstrate the need to adapt the sizes of footwear to the rapid increase in FL registered. Based on ours observations, and in coincidence with other authors, we propose changing footwear twice per year for children between 4 and 6 years of age, and three times per year for those between 6 and 12 years of age [3, 12]. This recommendation can suppose an impact on the family economy and to avoid these considerable financial implications, we agree with Barisch-Fritz et al. on the need to consider the use of 90th percentiles instead of mean values for the schoolchildren’s footwear manufacturing with adequate TA for both sexes [3]. We consider that footwear dynamic adjustments are applicable without customising to gender and age. However, it is important to account for the high variability and the different static and dynamic situations, with the purpose of improving footwear design and thus contribute to healthy foot development in children and adolescents.

As reinforcement for the study, the results regarding the quality of the anthropometric measurements made and especially the Inter-observer relative technical error of measurement (TEM %), have shown the reliability of the measurements made by the two operators who participated in the study. On the other hand, we highlight that we have considered the interior capacity of the footwear in the points where the foot presented the greatest dimensions, unlike other studies that measure the maximum interior capacity of the footwear without taking into account the different digital formulas. On the contrary, as a weakness, we point out the lack of a stop for the touch probe due to the non-resistance typical of the characteristics of the footwear’s material in the metatarsal area and of the upper. This circumstance may have generated some bias in the measurements results referring to height especially.

## Conclusions

The scarce evidence available makes it necessary to conduct prospective longitudinal studies to analyse the relevance that ill-fitting footwear during childhood may have on the future development of malformations in the adult foot. Thus, children’s footwear manufacturers will understand the need to design lasts from 3D technology adapted to the different foot morphologies, with the necessary allowances in length, width and height. On the other hand, when designing and selecting schoolchildren’s footwear size, it is also necessary to consider the biomechanical requirements of the children’s feet in primary education. We believe that the public administrations and the general population should be aware of the problem in order to find a solution. Meanwhile, public health practitioners should consider widespread implementation of school health programs that raise awareness among parents and teachers about the advisability of replacing periodically the footwear of schoolchildren within the age range of 5 to 12 years.

## Abbreviations

CV: Coefficient of variation
FH: Foot height
FL: Foot length
FW: Foot width
FWL: Footwear length
FWW: Footwear width
ICC: Intraclass correlation coefficient
TA: Toe allowance
TEM: Technical error measurement
